# Diagnostic challenges with accurate identification of *Listeria monocytogenes* isolates from food and environmental samples in South Africa

**DOI:** 10.4102/ajlm.v11i1.1482

**Published:** 2022-05-23

**Authors:** Teena S.M. Thomas, Juno Thomas, Karren le Roux, Sanelisiwe T. Duze, Faith Mkhwanazi, Adriano Duse

**Affiliations:** 1 Infection Control Services Laboratory, National Health Laboratory Services, Johannesburg, South Africa; 2 Department of Clinical Microbiology and Infectious Disease, School of Pathology, University of the Witwatersrand, Johannesburg, South Africa; 3 Centre for Enteric Diseases, National Institute of Communicable Diseases, Johannesburg, South Africa

**Keywords:** *Listeria monocytogenes*, food, environmental samples, diagnostic challenges, Africa

## Abstract

**Background:**

The 2017–2018 listeriosis outbreak in South Africa warranted testing for *Listeria monocytogenes* in food products and processing environments. Diagnostic tests are needed to accurately differentiate *L. monocytogenes* from other *Listeria* species.

**Objective:**

The study assessed the performance of the commonly used tests in our setting to accurately identify *L. monocytogenes*.

**Methods:**

The study was conducted in a public health laboratory in South Africa. Cultured isolates from food and environmental samples were tested both prospectively and retrospectively between August 2018 and December 2018. Isolates were phenotypically identified using tests for detecting β-haemolysis, Christie-Atkins-Munch-Peterson, alanine arylamidase (AlaA), mannosidase, and xylose fermentation. Listeria monocytogenes isolates were identified using automated systems, Microscan Walkaway Plus 96, Vitek® MS, Vitek^®^ 2 and Surefast *Listeria monocytogenes* PLUS PCR. All results were compared to whole-genome sequencing results.

**Results:**

β-haemolysis and Christie-Atkins-Munch-Peterson tests gave delayed positivity or were negative for *L. monocytogenes* and falsely positive for one strain of *Listeria innocua*. The AlaA enzyme and *Colorex Listeria* agar lacked specificity for *L. monocytogenes* identification. Based on a few phenotypic test results, an aberrant *L. monocytogenes* strain and *Listeria seeligeri* strain were reported. All automated platforms overcalled *L. monocytogenes* in place of other *Listeria* species.

**Conclusion:**

No test was ideal in differentiating *Listeria* species. This is an issue in resource-limited settings where these tests are currently used. Newer technologies based on enzyme-linked immunosorbent assay and other molecular techniques specific to *L. monocytogenes* detection need to be investigated.

## Introduction

An extensive outbreak of listeriosis occurred in South Africa in 2017–2018. To date, it is the largest laboratory-confirmed *Listeria monocytogenes* foodborne outbreak described globally.^1^ The *L. monocytogenes* strain responsible for the outbreak was characterised on whole-genome sequencing (WGS) analysis as multi-locus sequence type 6.^2^ Consequently, wide-scale testing for *L. monocytogenes* in various food products and processing environments commenced. For appropriate food safety and public health interventions, the utilised diagnostic tests should reliably differentiate *L. monocytogenes*, the outbreak pathogen, from other *Listeria* species. *Listeri*a species and *L. monocytogenes* share the same growth requirements and often coexist in the same environment; therefore, *L. monocytogenes* should be accurately discriminated from other co-occurring *Listeri*a species.^3^

Six *Listeria* species (*Listeria marthii, Listeria ivanovii, Listeria seeligeri, Listeria innocua, Listeria grayi* and *Listeria welshimeri*) are closely related to *L. monocytogenes*. This close-relatedness challenges species differentiation.^4^ Although uncommon, ‘atypical’ strains, which do not exhibit typical phenotypic characteristics, of *L. monocytogenes* and other *Listeria* species have also been described.^5^

Several test methodologies are utilised to discriminate between the *Listeria* species; however, each has its pitfalls. *L. monocytogenes* is positive for the Christie-Atkins-Munch-Peterson (CAMP) test on sheep blood agar within 24 h of incubation.^4^ However, weakly haemolytic (showing haemolysis beyond 24 h of incubation) or non-haemolytic strains are frequently detected. Weak or no haemolysis is due to deletion of the *hyl* gene or its regulatory protein *prfA*, which regulates the expression of virulence factors required for *L. monocytogenes* pathogenesis.^4^ Other *Listeria* species, such as *L. ivanovii* (particularly with the CAMP test utilising *Rhodococcus equi*), *L. seeligeri*, and some *L. innocua* strains also show haemolytic capabilities which can make the utility of this test pointless.^5,6,7^

*Listeria* agar by Ottaviani and Agosti (Agar *Listeria* Ottaviani & Agosti medium, BioRad, Berkeley, California, United States) is recommended in the International Organization for Standardization 11290–1, 2017 standard for the isolation and differentiation of *L. monocytogenes* from other *Listeria* species.^8^ All *Listeria* species are selected for growth on the medium and produce blue-green colonies due to substrate degradation by β-D-glucosidase activity. *L. monocytogenes* and *L. ivanovii* can be differentiated from the other species due to the production of an opaque halo around the colonies as a result of phosphatidylinositol-specific phospholipase (PI-PLC) activity.^9^ The timing of the appearance of the opaque halo is also indicative of the species type. The halo is produced after 24 h incubation by *L. monocytogenes* and after 48 h of incubation by *L. ivanovii*.^3,10^ Other strains, such as *L. seeligeri, L. welshimeri*, and a few strains of *L.* *innocua*, may also possess the *plcA* gene, which codes for phospholipase activity that is responsible for creating the opaque halo around its colonies.^4,5^ In addition, other bacterial species, like *Bacillus* species, *Cellulosimicrobium funkei*, enterococci, *Kochuria kristinae, Marinilactibacillus psychrotolerans, Rothia terrae*, and coagulase-negative staphylococci, may also grow as blue-green colonies on Agar *Listeria* Ottaviani & Agosti medium.^11^ *Bacillus circulans, Bacillus licheniformis, Enterococcus faecalis, Enterococcus faecium/durans*, and *Staphylococcus sciuri* can produce a halo as well, which can make differentiation of *L. monocytogenes* from *L.* *ivanovii* difficult.^11^

The Analytical Profile Index *Listeria* test (BioMerieux, Marcy d’Etoile, France) fails in 10% – 15% of identification cases. The main reason for this failure is due to the weak colour determinations. This is particularly applicable to the arylamidase test. The arylamidase enzyme, tested for in the popular Differentiation Innocua Monocytogenes (DIM) test, is supposed to be negative in *L. monocytogenes* and positive in other *Listeria* species.^12^ Often a weak positive DIM result was considered a negative result, increasing the false positive *L. monocytogenes* determinations.^4^ This might be due to the doubtfulness of the colour determinations by the reader of the test. Furthermore, false negative identification in atypical *L. monocytogenes* strains is also frequent.^4^

Matrix-Assisted Laser Desorption Time of Flight (MALDI-TOF) mass spectrometry is a quick and easy methodology gaining popularity in several microbiology laboratories. However, MALDI-TOF reportedly misidentifies *L. innocua* as *L. monocytogenes* American Type Culture Collection (ATCC) strain and the *L. seeligeri* ATCC strain as *L. monocytogenes* or *L. innocua*.^4^

Rychert et al. reported that Vitek^®^ Mass Spectrometer (MS) version 2.0 system (BioMerieux, Marcy d’Etoile, France) correctly identified only 76% (34/45) of *L. monocytogenes* to the species level and 9% (4/45) to the genus level, while in 15% (7/45) identification could not be finalised because split identification and re-testing were not performed.^13^ The Vitek^®^ 2 system has also been reported to misidentify *L. monocytogenes* as *L. innocu*a based on a negative reaction for phospholipase C in 1.4% (4/288) of a collection of isolates tested.^14^ The instrument could not identify an *L. monocytogenes* strain and gave a species error in another study.^15^ In a previous evaluation of the Vitek system, when genus level identification of various *Listeria* species was sought, the instrument had a sensitivity of 97.5%.^16^

The Microscan Walkaway Si system (Siemens Healthcare Diagnostics, West Sacramento, California, United States) could not identify one *L. monocytogenes* ATCC strain BAA–751 during a comparative study with the Vitek^®^ 2 compact system.^17^ The reason for this was potentially attributed to the limited number of *Listeria* species strains on the database.

During the investigation of the South African listeriosis outbreak in 2017–2018, four *Listeria* species (*L. monocytogenes, L. innocua, L. welshimeri*, and *L. seeligeri)* were detected from food samples and environmental swabs tested at the Infection Control Services Public Health Laboratory in Johannesburg, South Africa. This is similar to what has been described elsewhere in outbreak settings.^5^ As a result, accurate discrimination of *L. monocytogenes* from other species is of critical importance. Whole-genome sequencing is a useful tool for confirmatory identification of *L. monocytogenes* and can be used as the reference standard test for comparing other tests.^18^

Subsequent to the reported limitations of *Listeria* tests commonly utilised in most public health laboratories, particularly in low- and middle-income countries, the Infection Control Services Public Health Laboratory evaluated the performance of the commonly utilised phenotypic tests (conventional phenotypic tests and chromogenic media) for the identification of *Listeria* species in comparison to WGS results. The Infection Control Services Public Health Laboratory also compared the performance of the different automated diagnostic systems available in the institution for the identification of *L. monocytogenes* utilising known *Listeria* isolates characterised by WGS.

The study results will inform whether current tests are acceptable for future use and, if not, it will justify the evaluation of other technologies for accurate identification of *L. monocytogenes*.

## Methods

### Ethical considerations

Only cultured isolates from food and environmental samples were utilised in this research. No isolates from animals or animal-derived samples were used. Therefore, no ethical clearance was required.

### Study design and samples used

Data for this analysis were collected prospectively from August 2018 to December 2018 at the Infection Control Services Public Health Laboratory in Johannesburg. Isolates were cultured from food and environmental swabs of several food processing facilities across all of the provinces in South Africa during the listeriosis outbreak period. All *Listeria* isolates were identified on Vitek^®^ 2 (BioMerieux, Marcy-I’Etoile, France). The phenotypic tests were performed either to (1) confirm the initial identification from Vitek^®^ 2 or (2) to discriminate between *Listeria* species if two species identifications were given by Vitek^®^ 2. As a result, not all phenotypic tests were performed on all isolates. The accuracy of the conventional phenotypic tests to discriminate the four *Listeria* species (*L. monocytogenes, L. innocua, L. welshimeri*, and *L. seeligeri*) was assessed (Table 1).^3^ The isolate identity (Vitek^®^ 2 and phenotypic testing) was confirmed by WGS.

**TABLE 1:** Phenotypic tests used to discriminate *Listeria monocytogenes* from *L. innocua, L. welshimeri* and *L. seeligeri*.

Species	Βeta (β)-haemolysis (sheep blood agar plate)†	CAMP test (*S. aureus*†)	DIM test/AlaA‡	AMAN‡	dXYL‡
*L. monocytogenes*	+	+	–	+	–
*L. innocua*	–	–	+	+ (N/A)	(N/A)
*L. welshimeri*	–	–	V (N/A)	+ (N/A)	+
*L. seeligeri*	+ (N/A)	± (N/A)	+	–	+

*Source*: Adapted from Orsi RH, Wiedmann M. Characteristics and distribution of Listeria species including Listeria species newly described since 2009. Appl Microbiol Biotechnol. 2016;100(12):5273–5287. https://doi.org/10.1007/s00253-016-7552-2

AlaA, alanine arylamidase; AMAN, alpha (α)-mannosidase; CAMP, Christie, Atkins and Munch-Peterson; DIM, Differentiation Innocua Monocytogenes; dXYL, D-xylose; N/A, not applicable (as it does not assist in discriminating *L. monocytogenes* from the specific *Listeria* species); V, variable reactions.

† , At 24 h, 48 h or 72 h.

‡ , From Vitek 2 panel.

These isolates included 39 *L. monocytogenes* and 36 *Listeria* non-*monocytogenes* species, including 28 *L. innocua*, seven *L. welshimeri*, and one *L. seeligeri.*

### Laboratory analyses

Beta (β)-haemolysis was performed on sheep blood agar. Plates were checked daily for up to 72 h. The CAMP test was performed using the *Staphylococcus aureus* ATCC strain 25923. The positive controls used for this test were *Streptococcus agalactiae* ATCC 13813 and *L. monocytogenes* ATCC 19115. The plates were examined daily for up to 72 h. The presence of arylamidase, mannosidase enzymes and acid production from D-xylose (dXYL) fermentation was assessed on the Vitek^®^ 2 Gram-positive card based on the results of alanine arylamidase (AlaA), α-mannosidase (AMAN), and dXYL. The Vitek^®^ 2 Gram-positive card was inoculated with the isolates as per the Vitek^®^ 2 instrument training manual.^19^

The Colorex *Listeria* agar (E&O Laboratories Ltd, Bonnybridge, United Kingdom) has the same constituents as the Agar *Listeria* Ottaviani & Agosti medium. This medium was assessed and analysis was performed retrospectively using 59 of the 75 banked *Listeria* isolates from the analysis of the phenotypic tests. The isolates included 36 *L. monocytogenes*, 16 *L. innocua*, and seven *L. welshimeri*.

The laboratory also verified the performance of the automated platforms available. Analysis was performed retrospectively in December 2018 using 50 known *Listeria* isolates from the laboratory repository. The *Listeria* species included 20 *L. monocytogenes* strains and 30 *Listeria* species. The 30 *Listeria* species included 27 *L. innocua*, two *L. seeligeri*, and one *L. welshimeri.* These isolates were tested on four platforms, namely (1) Microscan Walkaway Plus 96 (Beckman Coulter Life Sciences, Indianapolis, Indiana, United States), (2) Vitek^®^ MS version 3.0 system (BioMerieux, Marcy-I’Etoile, France), (3) Vitek^®^ 2, and (4) Surefast *Listeria monocytogenes* PLUS polymerase chain reaction kit (Congen, Berlin, Germany), run on the Roche light cycler 2.0 (Roche, Basel, Switzerland) instrument. The Surefast kit identifies *L. monocytogenes* by amplifying a fragment of *prfA* and the detection limit of this assay is 10 CFU/mL as per our laboratory verification. Staff were blinded to the confirmatory WGS results of the isolates during the Colorex Listeria agar and automated systems assessments.

### Data analysis

The sensitivity, specificity, positive predictive value, and negative predictive value of the test methods for detecting *L. monocytogenes* were calculated. Data were collected on Excel spreadsheets (Microsoft, Redmond, Washington, United States) and analysis was performed using two-by-two tables.^20^

Calculations were done as follows:

- *Sensitivity* = True positive *L. monocytogenes* isolates (*Test and WGS positive*) / Total WGS-confirmed *L. monocytogenes* isolates (*True positive* + *False negative*) × 100
- *Specificity* = True negative *L. monocytogenes* isolates (*Test and WGS negative*) / Total WGS-confirmed non-*L. monocytogenes* isolates (*True negative* + *False positive*) × 100
- *Positive predictive value* = True positive *L. monocytogenes* isolates (*Test and WGS positive*) / Total positive test results (*True positive* + *False positive*) × 100
- *Negative predictive value* = True negative *L. monocytogenes* isolates (*Test and WGS negative*) / Total negative test results (*True negative* + *False negative*) × 100

## Results

The phenotypic results of the *Listeria* species in comparison to the WGS results are summarised in the Online Supplementary Table 1.

### Performance of the phenotypic tests for *L. monocytogenes* identification

The three phenotypic tests used to confirm the identification of *L. monocytogenes* by Vitek^®^ 2 and discriminate it from the other *Listeria* species were β-haemolysis, the CAMP test, and AlaA activity (Table 2).

**TABLE 2:** Performance characteristics of β-haemolysis, Christie, Atkins and Munch-Peterson test and Differentiation Innocua Monocytogenes test in comparison to whole-genome sequencing in the identification of *Listeria monocytogenes*, Infection Control Services Public Health Laboratory, South Africa, August 2018 – December 2018.

Phenotypic test	Performance indicators			
	Sensitivity (%)	Specificity (%)	Positive predictive value (%)	Negative predictive value (%)
Beta-haemolysis	82.0	94.0	94.0	82.9
Christie, Atkins and Munch-Peterson test	82.0	94.0	94.0	82.9
DIM test/AlaA activity	81.6	0.0	51.6	0.0

DIM, Differentiation Innocua Monocytogenes; AlaA, alanine arylamidase.

Of the 39 *L. monocytogenes* isolates identified by WGS, all three phenotypic tests corroborated the WGS findings in 82% (32/39) of the isolates. β-haemolysis and the CAMP test were absent in 18% (7/39) of the isolates. Delayed positivity to both of these tests occurred at 72 h in 5.1% (2/39) of isolates. One *L. innocua* isolate was falsely positive to both of these tests.

Of the *L. monocytogenes* isolates, 18% (7/39) were falsely positive for AlaA on Vitek^®^ 2. All *L. innocua* isolates and the one *L. seeligeri* isolate were falsely negative for AlaA.

### Performance of the phenotypic tests for *L. innocua* identification

Of note, one out of the 28 *L. innocua* isolates was positive for both β-haemolysis and the CAMP test.

### Performance of the phenotypic tests for *L. welshimeri* identification

The phenotypic tests used to identify *L. welshimeri* identified all seven of the isolates correctly. However, there was one isolate that had two identification options on Vitek^®^ 2, namely *L. monocytogenes* and *L. welshimeri.* To discriminate between the two *Listeria* species, β-haemolysis, CAMP, and dXYL fermentation tests were assessed. The isolate was negative for β-haemolysis and the CAMP test but positive for dXYL fermentation, which suggested that the isolate is *L. welshimeri*. However, WGS results identified the isolate as *L. monocytogenes.* Performance indicators, such as sensitivity, specificity, positive predictive value, and negative predictive value, for the phenotypic tests (β-haemolysis, CAMP test and dXYL fermentation) differentiating *L. welshimeri* from *L. monocytogenes* were not done due to the low number of *L. welshimeri* isolates identified during the study period.

### Performance of the phenotypic tests for *L. seeligeri* identification

One isolate was previously identified as *L. monocytogenes* based on Vitek^®^ 2 identification (99% probability), positive β-haemolysis (at 24 h incubation), positive CAMP test (at 24 h incubation), negative AlaA enzyme activity, negative dXYL fermentation, and positive AMAN activity. However, WGS identified this isolate as *L. seeligeri.* Since there was only one *L. seeligeri* isolate, the performance of the tests (AlaA activity, AMAN activity, and dXYL fermentation) to discriminate this species from *L. monocytogenes* was not done.

### Performance of the Colorex *Listeria* agar in the identification of *L. monocytogenes*

**FIGURE 1:**
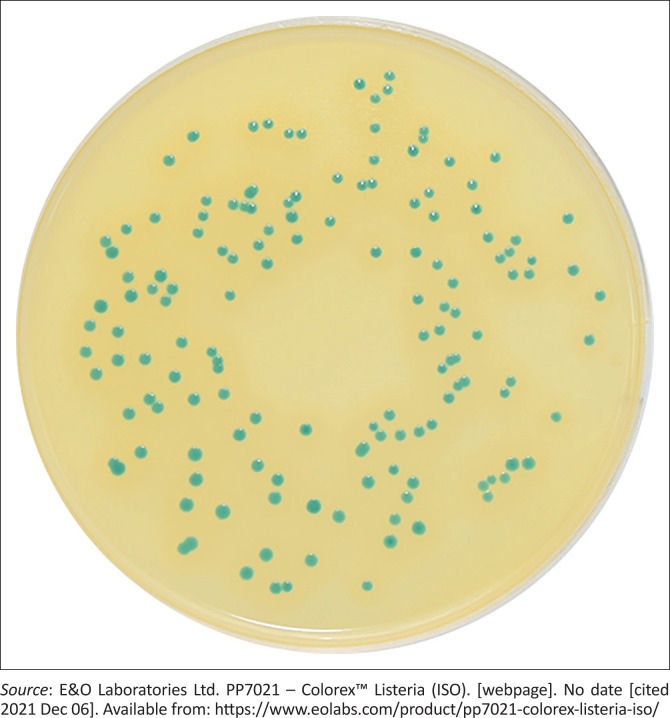
*Listeria monocytogenes* colonies on Colorex *Listeria* Agar.

All 59 isolates representing the three *Listeria* species produced blue-green colonies on Colorex *Listeria* agar and 73% (43/59) of these isolates produced an opaque halo around the colonies (Figure 1).^21^ Of the 43 isolates that produced a halo, 84% (36/43) were identified as *L. monocytogenes* on WGS, while the remaining 16% (7/43) of isolates were identified as *L. innocua* (*n* = 5) and *L. welshimeri* (*n* = 2) on WGS (Online Supplementary Table 2). The sensitivity and specificity of the medium for accurate *L. monocytogenes* identification were 100% and 69%. The positive predictive value was 83.7% and negative predictive value was 100%.

### Performance of the automated systems in the identification of *L. monocytogenes*

All the systems overcalled *L. monocytogenes* in place of other species (Table 3). All *Listeria* species results on the various automated systems are provided in Online Supplementary Table 3.

**TABLE 3:** Performance characteristics of the automated systems in comparison to whole-genome sequencing in the identification of *Listeria monocytogenes*, Infection Control Services Public Health Laboratory, South Africa, December 2018.

Automated tests	Performance indicators				Comments
	Sensitivity (%)	Specificity (%)	Positive predictive value (%)	Negative predictive value (%)	
Microscan (Beckman & Coulter)	100	6.6	41.6	100	Overcalls *L. monocytogenes* for *L. innocua* (*n* = 27) & *L. welshimeri* (*n* = 1)
Vitek^®^ mass spectrometry (BioMerieux)	80	56.6	55.1	81	Overcalls *L. monocytogenes* for *L. innocua* (*n* = 11) & *L. seeligeri* (*n* = 2). *L. monocytogenes* misidentified as *L. innocua* (*n* = 3) & *L. welshimeri* (*n* = 1)
Vitek^®^ 2 (BioMerieux)	100	76.6	74	100	Overcalls *L. monocytogenes* for *L. innocua* (*n* = 7). In 6/7 of the isolates, Vitek 2 gave both identifications & suggested discrimination with beta-haemolysis & the Christie, Atkins and Munch-Peterson test
PCR (Surefast kit, Congen)	100	93.3	91	100	Overcalls *L. monocytogenes* with *L. innocua* (*n* = 2)

PCR, polymerase chain reaction.

## Discussion

From the evaluation, there was no ideal test for differentiating the *Listeria* species: all had limitations. β-haemolysis and the CAMP test are recommended to differentiate *L. monocytogenes* from *L. innocua*. However, these tests can give delayed positivity (up to three days later) or be negative for *L. monocytogenes.* In addition, they may be falsely positive for certain *L. innocua* strains. The DIM test for AlaA enzyme activity lacks specificity for *L. monocytogenes* detection. All of the other *Listeria* species also tested negative for this enzymatic activity, disproving its utility.

We report the first aberrant *L. monocytogenes* strain that fermented dXYL and an aberrant *L. seeligeri* strain that was negative for AlaA activity and dXYL fermentation and positive for AMAN activity. This further illustrates atypical strains that may potentially exist in our setting, complicating identification. Unfortunately, WGS could not be repeated on both of these isolates again to reconfirm the results and rule out the possibility of isolate mix-up.

The Colorex *Listeria* agar was able to correctly identify all *L. monocytogenes* isolates; however, it lacked specificity in discriminating other *Listeria* species from *L. monocytogenes*.

### Limitations

There were also several shortcomings associated with the four automated diagnostic platforms tested. All platforms overcalled *L. monocytogenes* in place of the other *Listeria* species. This could be because these instruments were validated for clinical samples in which *L. monocytogenes* is the predominant species isolated. The Vitek^®^ MS misidentified *L. monocytogenes* for other *Listeria* species and the Vitek^®^ 2 gave both *L. monocytogenes* and *L. innocua* options for a few *L. innocua* isolates. The worst-performing platform for *L. monocytogenes* identification was Microscan and the best performer was the Surefast polymerase chain reaction kit.

Based on the above results, alternate testing platforms for *L. monocytogenes* identification need to be investigated.

Several other diagnostic methodologies are available to detect *L. monocytogenes*. These include (1) detection of *L. monocytogenes* by antibody-based assays, (2) molecular test methods such as Loop-mediated isothermal amplification (LAMP), DNA hybridisation or polymerase chain reaction utilising *L. monocytogenes*–specific gene targets that have been identified, and (3) the use of genetically engineered bacteriophages.^3,7,9,12^ Many of these assays are available as commercial automated kits approved by regulatory authorities. These technologies have been reported to perform well in the detection of *L. monocytogenes*. In addition, the automated systems are high throughput and significantly shortens the time to results of the traditional methods. The possible disadvantages of the above technologies would be: cost, staff expertise to perform the tests, and inhibition of tests (antibody-based and molecular) by the sample matrix. The molecular assays may also detect non-viable organisms.

### Conclusion

We have demonstrated that the commonly used methodologies in most public health laboratories, particularly in low- and middle-income settings, are limited in differentiating *L. monocytogenes* from the other *Listeri*a species. The accurate identification of *L. monocytogenes* is critical since it is the most predominant *Listeria* species causing human disease and, therefore, must not be missed by diagnostic tests. The large scale of this outbreak required upscaling laboratory support for public health sample testing. However, if the available systems in routine microbiology laboratories cannot discriminate between *L. monocytogenes* and the other *Listeria* species, overcalling or underreporting of *L. monocytogenes* can occur. Underreporting *L. monocytogenes* will prevent or delay identifying an outbreak source and promote its continuity with huge public health impact. Overcalling *L. monocytogenes* leads to the unnecessary closure of food production lines, which has huge financial implications for the company involved. Depending on the company’s distribution level, halting production can also impact the community. In the outbreak setting, where *L. monocytogenes* prevalence in samples was comparatively higher, the positive predictive value of most of the tests assessed was unacceptable. Hence, in a non-outbreak setting, the performance of these tests will be worse.

Therefore, other technologies must be investigated for their discriminatory capabilities and accurate identification of *L. monocytogenes* in microbiology laboratories in low- and middle-income countries.
